# Ameliorating Effect of *Akebia quinata* Fruit Extracts on Skin Aging Induced by Advanced Glycation End Products

**DOI:** 10.3390/nu7115478

**Published:** 2015-11-12

**Authors:** Seoungwoo Shin, Dahee Son, Minkyung Kim, Seungjun Lee, Kyung-Baeg Roh, Dehun Ryu, Jongsung Lee, Eunsun Jung, Deokhoon Park

**Affiliations:** 1 Biospectrum Life Science Institute, Eines Platz 11th FL, 442-13 Sangdaewon Dong, Seoungnam City, Gyunggi Do 462-807, Korea; biost@biospectrum.com (S.S.); biouk@biospectrum.com (D.S.); biotq@biospectrum.com (M.K.); biotf@biospectrum.com (S.L.); biosh@biospectrum.com (K.-B.R.); biosc@biospectrum.com (D.R.); pdh@biospectrum.com (D.P.); 2 Department of Genetic Engineering, Sungkyunkwan University, 2066, Seobu-Ro, Jangan Gu, Suwon City, Gyunggi Do 164-19, Korea

**Keywords:** AGE, fibrillin-1, glycation, human skin explants, *Akebia quinata* fruit extract

## Abstract

The accumulation of free radicals and advanced glycation end products (AGEs) in the skin plays a very important role in skin aging. Both are known to interact with each other. Therefore, natural compounds or extracts that possess both antioxidant and antiglycation activities might have great antiageing potential. *Akebia quinata* fruit extract (AQFE) has been used to treat urinary tract inflammatory disease in traditional Korean and Chinese medicines. In the present study, AQFE was demonstrated to possess antioxidant and antiglycation activity. AQFE protects human dermal fibroblasts (HDFs) from oxidative stress and inhibits cellular senescence induced by oxidative stress. We also found that AQFE inhibits glycation reaction between BSA and glucose. The antiglycation activity of AQFE was dose-dependent. In addition, the antiglycation activity of AQFE was confirmed in a human skin explant model. AQFE reduced CML expression and stimulated fibrillin-1 expression in comparison to the methyglyoxal treatment. In addition, the possibility of the extract as an anti-skin aging agent has also been clinically validated. Our analysis of the crow’s feet wrinkle showed that there was a decrease in the depth of deep furrows in RI treated with AQFE cream over an eight-week period. The overall results suggest that AQFE may work as an anti-skin aging agent by preventing oxidative stress and other complications associated with AGEs formation.

## 1. Introduction

In recent years, the role of advanced glycation end products (AGEs) has been increasingly discussed in skin aging, and the potential of anti-AGE strategies has received high interest from pharmaceutical companies for the development of novel antiaging cosmeceutical compounds.

Glycation is a slow, nonenzymatic reaction that takes place between free amino groups in proteins primarily lysine, and a reducing sugar such as glucose or ribose. In skin, this reaction creates new residues or formations of cross-links (AGEs) in the extracellular matrix of the dermis. Proteins like collagen with a long half-life and a slow turnover can be affected by this phenomenon *in vivo* [1]. Glycation reactions are characterized by the presence of carboxymethyl-lysine (CML) and pentosidine, which are AGEs [2]. AGEs markedly increase in diabetes, due to a higher glycolytic rate, and settle on the elastic network. They arise from dicarbonyl precursors, a category among which methyglyoxal can be included. Among the other markers studied for their sensitivity to glycation, it was shown that fibrillin-1, a glycoprotein associated with oxytalan fibers, is highly sensitive to glycation and its alteration was inversely correlated to the appearance of CML [3]. Fibrillin-1 contributes to the clinical features of skin aging, such as wrinkle formation and loss of elasticity [4].

The accumulation of AGEs with age is well-known, notably in skin, and is believed to modify skin homeostasis [5]. For example, AGEs have been shown to alter fibroblast viability by inducing the apoptotic process [6,7], a senescence phenotype [8] or a cytotoxic effect [9]. Glycation products have also been reported to alter the biology of these dermal cells by disturbing the balance between the synthesis of extracellular matrix components and enzymes [10,11,12] and, consequently, skin structure. Most of these biological responses can be mediated by the Receptor of AGE (RAGE). AGE–RAGE interaction alters cellular signaling, alters gene expression, enhances the release of pro-inflammatory cytokines and increases intracellular reactive oxygen species (ROS) via NADPH-oxidase [13]. If biological signature is altered, mechanical properties are also disrupted [14] and could explain the loss of elasticity observed with age [15]. Glycation may therefore play an important role in chronologic aging. In recent years, research exploring the anti-AGE activities of natural foods has attracted much attention in Korea as well as in some other Asian countries, and some very promising results have been obtained, e.g., from mung bean [16], tomato paste [17], guava [18], and microalgae [19,20]. These findings, with a few exceptions, highly correlate with free radical scavenging activities, providing very good evidence for the management of skin aging by antiaging therapy. We are interested in the screening and development of antioxidants containing significant antiglycative capacities from natural sources because natural products have fewer side effects and are much safer for human consumption than synthetic agents.

*Akebia quinata* (Thunb.) Decne. (*A. quinata*) is a creeping woody vine that is widely distributed in East Asia. The dry ripe fruit and stem of *A. quinata* is used as an analgesic, an antiphlogistic, and a diuretic in traditional Chinese medicine [21]. Furthermore, *A. quinata* is used as a crude drug material for treating obesity in traditional Korean medicine [22]. The dried fruits, leaves, and stem of the plant are also ingredients of a traditional Korean weight-loss tea used as a folk remedy. The *A. quinata* extract has been shown to have antioxidant activity and free radical scavenging capability [23]. However, until now, there have been very few report which show chemical composition of AQFE. Some studies just reported that *Akebia quinata* extract contained chlorogenic acid, isochlorogenic acid A, isochlorogenic acid C, triterpenoid saponins [22,24]. In addition, other possible novel functions of *A. quinata* extract in skin biology remain elusive.

This study aimed to investigate the anti-skin aging potential of AQFE *in vitro* and in the living human skin explants model. We also confirmed the anti-skin aging effect of AQFE in a clinical study.

## 2. Experimental Section

### 2.1. Chemicals and Antibodies

Bovine serum albumin (BSA, fraction V), d-glucose, aminoguanidine (AG), and 2,4-dinitrophenylhydrazine (DNPH) were obtained from Sigma (St. Louis, MO, USA). Trichloroacetic acid (TCA) and guanidine hydrochloride were purchased from Merck (Darmstadt, Germany). OxiSelect™ N^ε^-CML ELISA kit was purchased from Cell Biolabs (San Diego, CA, USA). All other chemicals used were of analytical grade.

### 2.2. Plant Preparation and Extraction

The fruit of *Akebia quinta* was purchased from Jeju (Korea). The powdered *Akebia quinta* fruit was extracted with 70% ethanol. To prepare the ethanol extract, powdered *Akebia quinta* fruit (70.9 g) was extracted overnight with 70% ethanol (5 L) at room temperature with stirring. The supernatant was collected by filtration through filter paper. Ethanol was removed by rotary vacuum evaporator (EYELA, Tokyo, Japan) and the extract was lyophilized (12.2 g).

### 2.3. In Vitro Glycation of Bovine Serum Albumin (BSA)

The preparation of glycated BSA was performed according to a previously described method with minor modifications [25]. BSA (10 mg/mL) was incubated for three weeks at 37 °C with glucose (0.5 M) in 0.1 M phosphate buffer (PBS) at a pH of 7.4. The solution contained 0.02% sodium azide (NaN_3_) and the incubation was conducted in the absence or presence of AQFE (10–500 µg/mL) and aminoguanidine (500 µg/mL). Aliquots of the reaction mixtures were then assayed for AGEs formation, and CML.

### 2.4. Determination of AGEs Formation

The formation of AGEs was determined spectrofluorometrically (Infinite^®^ F200 Pro, Tecan group Ltd., Männedorf, Switzerland) at excitation and emission wavelengths of 340 and 430 nm, respectively. The inhibitory effect of AQFE and aminoguanidine was evaluated by calculating the percentage inhibition compared with the maximum glycation elicited by glucose. The percentage of fluorescent AGE formation was calculated as follows:

Inhibition of fluorescent AGEs (%) = ((F_C_ − F_CB_) − (F_S_ − F_SB_)/(F_C_ − F_CB_)) × 100

where F_C_ and F_CB_ were the fluorescent intensity of the control with glucose and the blank image from the control without glucose, F_S_ and F_SB_ were the fluorescent intensity of the sample with glucose and the blank image from the sample without glucose.

### 2.5. Determination of N^ε^-CML

N^ε^-CML, a major antigenic AGE structure, was determined using an ELISA kit according to the manufacturer’s protocol. The absorbance of samples was compared with the CML-BSA standard provided in the assay kit.

### 2.6. Cell Line and Cell Culture

Human dermal fibroblasts (derived from neonatal foreskin) were purchased from American Type Cell Collection (ATCC). The cells were cultured in Dulbecco Modified Eagle Medium containing 10% fetal bovine serum (Gibco-BRL, Sparks, MD, USA) and penicillin streptomycin at 37 °C in a humidified atmosphere containing 95% air/5% carbon dioxide [26].

### 2.7. DPPH Scavenging Activity Assay

The antioxidant activity of AQFE was first determined by measuring the DPPH scavenging ability [27]. The extract at various concentrations (25–500 μg/mL) was added to 200 μL of DPPH (100 μM) solution. DPPH reaction with an antioxidant capable of hydrogen ion donation reduces DPPH. The resulting decrease in absorbance at 540 nm was recorded using a Gen 5™ UV-Vis spectrophotometer (BioTek, Winooski, VT, USA).

### 2.8. ABTS^+^ Scavenging Capacity Assay

The ABTS decolorization assay was performed as previously described [28]. The assay relies on the generation of ABTS^+^ chromophore by the oxidation of ABTS with potassium persulfate. The ABTS radical cation (ABTS^+^) was produced by reacting 7 mM stock solution of ABTS with 2.45 mM potassium persulfate and allowing the mixture to stand in the dark for at least 6 h before use. The absorbance at 734 nm was measured 30 min after the mixing of various concentrations of AQFE (25−500 μg/mL) with 1 mL of ABTS^+^ solution.

### 2.9. Determination of Ferric Reducing Capacity

The ferric reducing antioxidant power (FRAP) of the extract was determined as previously described [29]. The FRAP reagent was produced by mixing 300 mM acetate buffer (pH 3.6), 10 mM TPTZ solution, and 20 mM FeCl_3_⋅6H_2_O in a 10:1:1 ratio and was freshly prepared at 37 °C. Different concentrations of AQFE (25~500 μg/mL) were individually mixed with FRAP reagent. The mixture was incubated at room temperature for 30 min in the dark. The absorbance was measured at 595 nm in the UV-Vis spectrophotometer. Higher absorbance of the reaction mixture indicated greater reducing power.

### 2.10. Intracellular ROS Measurement

Intracellular ROS levels were determined by measuring the DCFH-DA as previously described [30]. Briefly, cells pre-loaded with 100 μM H_2_DCFDA (Molecular Probe, Eugene, OR, USA) at 37 °C in the dark for 30 min were exposed to AGE-BSA, and were then lysed in 1 N NaOH. The fluorescence intensity of each lysate was measured at an excitation wavelength of 485 nm and an emission wavelength of 535 nm using a fluorometer (INFINITE M200, Tecan, Männedorf, Switzerland). The intracellular accumulation of ROS was also observed under an Evos fluorescent microscope (Advanced Microscopy Group, Bothell, WA, USA). Nonglycated BSA was used as a control.

### 2.11. Senescence-Associated Galactosidase (SA-β-Gal) Activity

A commercial senescence-associated β-galactosidase kit (SA-β-gal, Sigma) was used to measure β-galactosidase activity, which is characteristic of senescent cells [31]. SA-β-gal is used to assess the abnormal enzymatic activity observed in aging. The culture was incubated for two hours after adding a staining solution; β-galactosidase in senescent cells stains blue. The proportion of senescent cells was computed as the number of senescent cells stained blue divided by the total number of cells. A round optical glass with a grid dividing the view into five square fields was attached to the eyepiece of an optical microscope and used to count the number of cells in each field over ten fields. The average number of stained and total cells was determined for these ten fields (×100).

### 2.12. Human Skin Explants

The first investigations refer to the biological activity of a carboxymethyl cellulose gel containing 1% AQFE on live human skin explants. The study was performed in accordance with the Declaration of Helsinki after the patient had given informed consent. The explants were obtained as full thickness human skin biopsies from an abdominoplasty of a 34-year-old Caucasian woman. Punch biopsies removed from the patient were immediately sent to Laboratoire BIO-EC (Longjumeau, France), where the hypodermis was removed from the skin and circular samples were excised using a punch instrument. The samples, with the dermis face down, were immediately placed in a liquid-air interface in Laboratoire BIO-EC’s Explant Medium (BEM^®^) and cultured under classical cell-culture conditions (37 °C in 5% CO_2_). Half of the medium (1 mL) was refreshed every other day. The general cells morphology was evaluated prior to all other instrumental observations.

### 2.13. Antiglycation Activity in Human Skin Explants

AQFE was applied to 18 live human skin explants with an average diameter of 11 mm. The explants were prepared from an abdominoplasty of a 34-year-old Caucasian woman. The explants were kept alive in BEM culture medium at 37 °C in a humid, 5% CO_2_ atmosphere. The applied product was a carboxymethyl cellulose gel with a final concentration of 1% and the application amount was 2 mg/explant. Methylglyoxal (MG) was applied as a glycation promoter at 500 µM on days 3, 5, and 7. Observation of the general morphology was performed after staining paraffinized sections according to Masson’s trichrome method, using the Goldner variant. The immunostaining of fibrillin-1 was performed on frozen sections with an anti-fibrillin-1 antibody, clone 11C1.3 (NB110-8146, Novus Biologicals, Littleton, CO, USA). The antibody was applied for 1 h at room temperature with a biotin/streptavidin enhancement system (Vector, Burlingame, CA, USA, Vectastain) and revealed by fluorescein isothiocyanate (FITC). Nuclei were counterstained with propidium iodide. The immunostaining for CML was performed on a paraffinized section with an anti-CML antibody, clone CMS-10-12 (Trans Genic Inc., Kobe, Japan, ref KH011). The antibody was applied for one night at room temperature using a biotin/streptavidin enhancement system (Vector, Vectastain) and revealed by VIP (dark puple) chromagen substrate (Vector, SK-4600).

General morphology, and CML immunostaining were observed using a Leica optical microscope type DMLB at the magnification of 40×. Fibrillin-1 was observed using a fluorescence microscope type DMLB at the magnification of 40×. Photos were taken with a numeric DP72 Olympus camera and stored with the CellD storing software. At least 3 explants were analyzed in each group. The explants were distributed in six batches as follows: Control (D8), no treatment, sampling on day 8; AG (D8), treatment with aminoguanidine 1% solution, sampling on day 8; AQFE (D8), treatment with AQFE 1% solution, sampling on day 8; MG (D8), treatment with MG, sampling on day 8; MG + AG (D8), treatment with both aminoguanidine 1% solution and MG, sampling on day 8, and MG + AQFE (D8), treatment with both AQFE 1% solution and MG, sampling on day 8.

Planimetry analysis of the positive staining signals was performed using Image J software, as described previously [32]. The area of positive staining divided by the total area was calculated. Each value presented is expressed as the mean ± standard deviation, and all quantitative results represent at least three independent analyses.

### 2.14. Image Analysis of Cow’s Feet Wrinkle

Volunteers were selected according to previously agreed inclusion criteria (age 30–60 years) and signed the informed consent form (in compliance with the Declaration of Helsinki and the 1988 Act of the Code de la Santé Publique, France) prior to the beginning of the study on day 0. This study was approved by the ethics committee of the DERMAPRO/Skin Research Center, and subjects gave written informed consent. They were stabilized in a controlled room atmosphere for 10 min prior to all basal measurements. Seventeen healthy subjects (average age: 47.47 ± 3.32 year) with crow’s feet participated in this study. Visual assessment and skin wrinkle parameters for crow’s feet were measured at the first visit (base line), and at 4 and 8 weeks following 8 consecutive weeks of treatment with the test product. Skin wrinkles at the test site (crow’s feet area in subjects) were evaluated using 3D Image Analysis GFM PRIMOS (GFMesstechnik GmbH, Berlin, Germany). Before treatment and at 4 and 8 weeks after treatment, 4 wrinkle parameters (Ra: arithmetic average roughness; Rmax: maximum roughness; Rz: average roughness; Rp: smoothness depth) were analyzed. All obtained data were analyzed by a paired *t*-test in the SPSS 11.5 package program. This study was approved by the ethics committee of the DERMAPRO/Skin Research Center (Seoul, Korea), and subjects provided written informed consent.

### 2.15. Statistical Analysis

All data are expressed as mean ± SD. Differences between the control and treatment groups were evaluated by one way ANOVA using SPSS software, version 22.0 (IBM Corporation, New York, NY, USA). A *p* < 0.01 was considered statistically significant.

## 3. Results and Discussion

### 3.1. The Effect of AQFE on AGEs Formation

**Figure 1:**
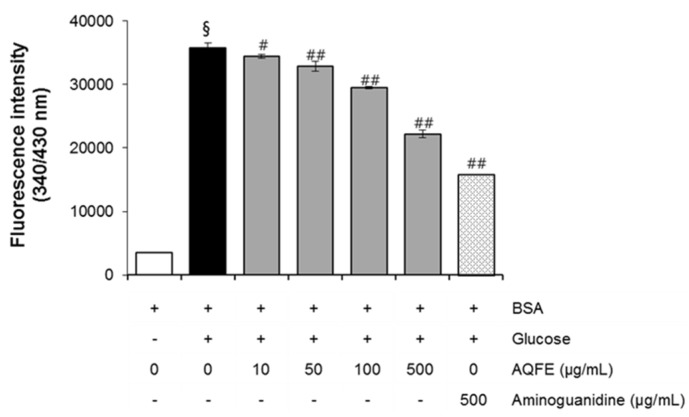
The effects of AQFE on formation of fluorescent advanced glycation end products in BSA incubated with glucose. Data are mean ± standard deviation. ^§^ *p* < 0.01 compared with the vehicle-treated group; ^#^ *p* < 0.05 compared with the BSA/glucose treated group; ^##^ *p* < 0.01 compared with the BSA/glucose treated group (*n* = 5). The results were confirmed by eight independent experiments.

In the present study, the AGE inhibitory capacities of AQFE were evaluated for the first time. The BSA-glucose system employed is commonly used in non-enzymatic glycation studies. In the BSA-glucose system, AQFE exhibited significant AGE inhibitory ability in a dose-dependent manner. As shown in Figure 1, the formation of AGEs was monitored at Week 3 by measuring the fluorescence intensity of the BSA-glucose solutions. When BSA was incubated with glucose, a significant increase in fluorescence intensity was observed at Week 3 of the experiment. After AQFE was added to the reaction media containing BSA/glucose, the fluorescence intensity decreased significantly in a concentration-dependent manner throughout the study period. At Week 3 of incubation, the percentage inhibition of AGEs formation by AQFE (10–500 µg/mL) was 3.0% to 37.4%, respectively.

### 3.2. Topical Antiglycation Activity of AQFE

On an abdominoplasty, donated by a 34-year-old Caucasian woman, aminoguanidine and AQFE were incorporated in a neutral gel at a dose of 1% and applied topically to the surface of the explants for eight days. After three days of treatment, methylglyoxal was added to the culture medium.

**Figure 2:**
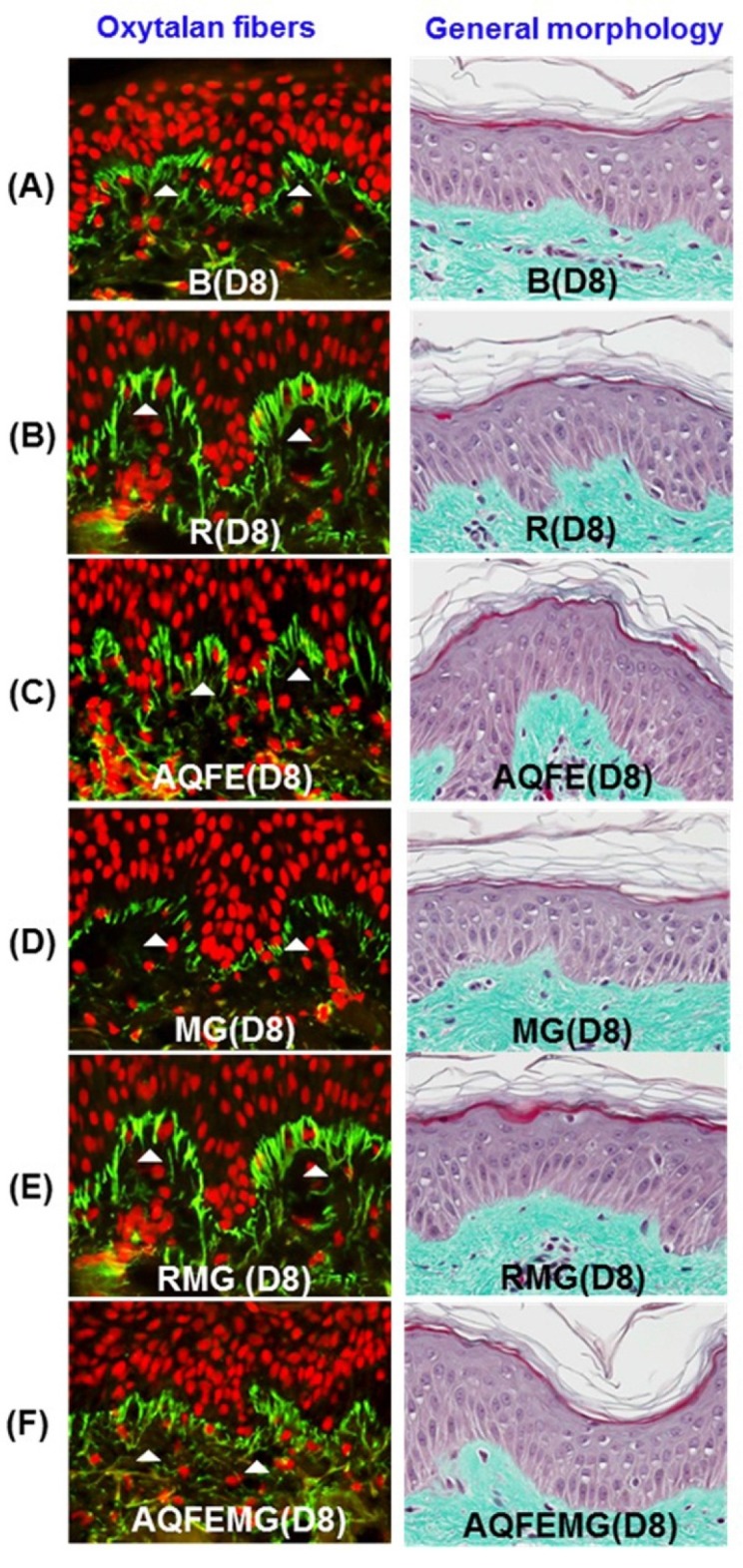

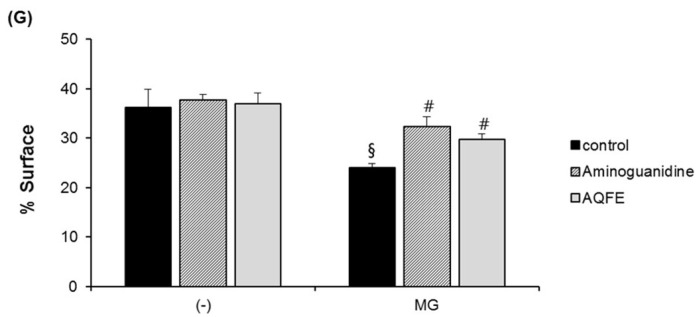
The explants were kept alive in a BEM culture medium for eight days with the vehicle and 1% AQFE. Fibrillin-1 immunostaining was conducted. Immunostaining of fibrillin-1 on untreated batch (**A**); treated with AG (**B**); treated with AQFE (**C**); treated with MG (**D**); treated with MG + AG (**E**) and treated with MG + AQFE (**F**). (**G**) Image analysis of the surface percentage occupied by fibrillin-1 under the dermal-epidermal junction, as a function of the product applied. The average fluorescence intensity values were calculated using Image J software. Data are mean ± standard deviation. ^§^ *p* < 0.01 compared with the vehicle-treated group; ^#^ *p* < 0.05 compared with the MG-treated group (*n* = 3). The results were confirmed by eight independent experiments.

When applied topically to glycated explants, aminoguanidine showed the same protective effect on fibrillin-1 as when added to the culture medium in the second step. The fibrillin-1 network was unchanged compared to the control batch and was protected from methylglyoxal-induced glycation (Figure 2). The degradation of fibrillin-1 through methylglyoxal-induced glycation was clearly highlighted, with a decrease of 26.7% in the surface percentage occupied by fibrillin-1 under the dermal-epidermal junction (DEJ), compared to the untreated control batch. The AQFE applied topically also protected fibrillin-1 from glycation and the fibrillin-1 staining was similar to the control batch without induced glycation (Figure 2A–F). These findings were confirmed by the results obtained from the image analysis of fibrillin-1, presented in Figure 2G.

**Figure 3:**
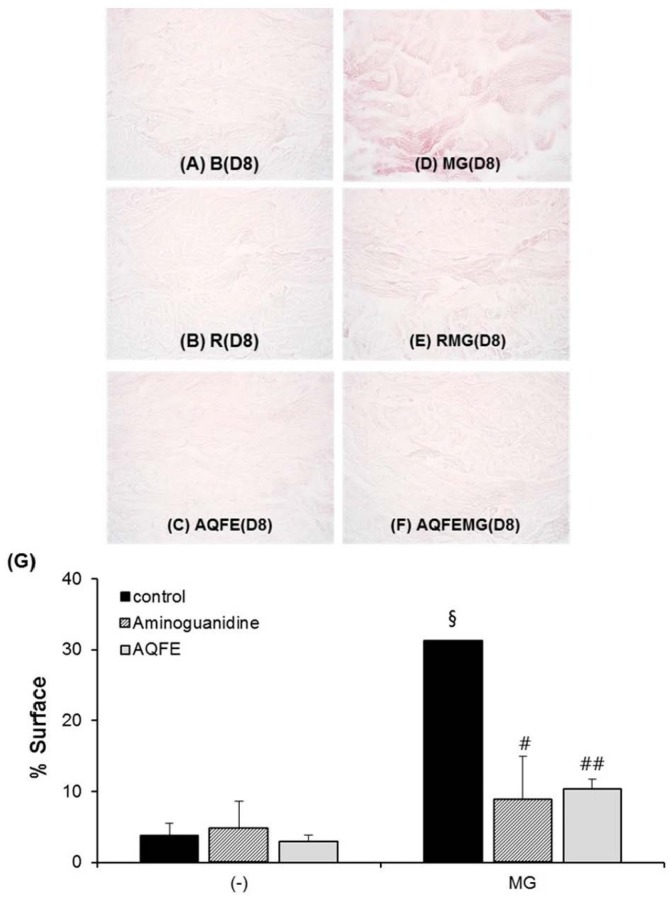
Activity of AQFE against methylglyoxal-induced glycation, revealed by the immunostaining of CML. The explants were kept alive in a BEM culture medium for eight days with the vehicle and 1% AQFE. CML immunostaining was conducted. Immunostaining of CML on untreated batch (**A**); treated with AG (**B**); treated with AQFE (**C**); treated with MG (**D**); treated with MG + AG (**E**) and treated with MG + AQFE (**F**). (**G**) Staining intensity on batches treated with active ingredients on CML immunostaining. The average staining intensity values were calculated using Image J software. Data are mean ± standard deviation. ^§^ *p* < 0.01 compared with the vehicle-treated group; ^#^ *p* < 0.05 compared with the MG-treated group; ^##^ *p* < 0.01 compared with the MG-treated group (*n* = 3). The results were confirmed by eight independent experiments.

A decrease in the staining of CML (Figure 3A–F) was also observed in the glycated explants treated with AG and AQFE unlike the explants treated with MG. MG induced a clear increase in CML expression on Day 8. The AQFE-treated batch (not stimulated with MG) showed complete inhibition of physiological CML expression. The AQFE-treated batch induced by MG showed complete inhibition of CML expression induced by MG. AQFE showed a clear inhibition of CML expression in both the MG-treated and non-MG-treated batches. These findings were confirmed by the results obtained from the image analysis of CML, presented in Figure 3G.

### 3.3. Antioxidant Capacities of AQFE

**Figure 4:**
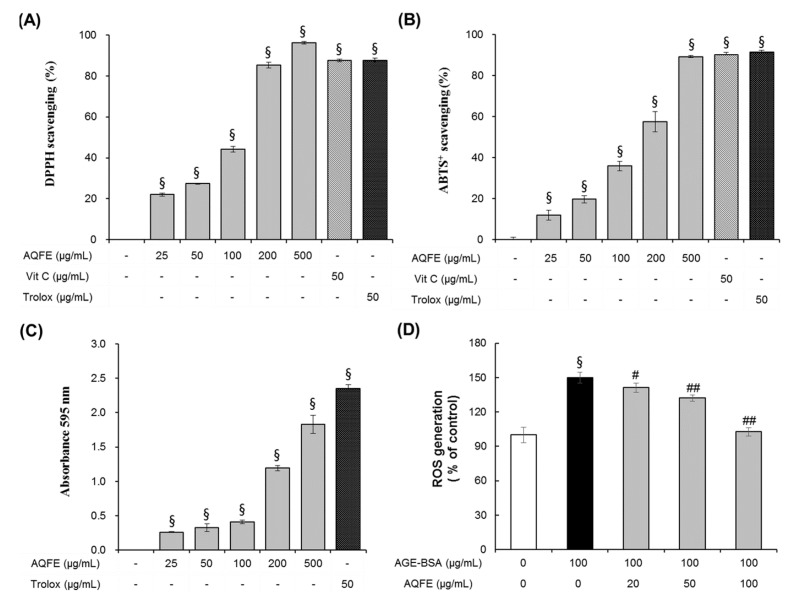
The antioxidant properties of AQFE. Radical scavenging activity was determined by using different concentrations of AQFE (25–500 μg/mL) or vitamin C and trolox through DPPH (**A**) and ABTS^+^ (**B**) assays. The effect of AQFE was confirmed by measurement of reducing capacity (**C**). Effect of AQFE on intracellular ROS production in AGE-BSA treated human dermal fibroblast cells (**D**). Data are mean ± standard deviation. ^§^ *p* < 0.01 compared with the vehicle-treated group, ^#^ *p* < 0.05 compared with the AGE-BSA treated group, ^##^ *p* < 0.01 compared with the AGE-BSA treated group (*n* = 5). The results were confirmed by eight independent experiments.

In the present work, several assays were employed to evaluate the antioxidant activity of AQFE (Figure 4). Radical-scavenging activity was assessed by the DPPH assay using vitamin C (50 μg/mL) and trolox (50 μg/mL) as comparative controls. DPPH scavenging activity of the various concentrations of AQFE (25–500 μg/mL) ranged from 22.10% ± 0.76% to 97.86% ± 0.00%, with an IC_50_ of 112.04 ± 0.40 μg/mL (Figure 4A). The results confirmed free radical-scavenging activity by AQFE in a dose-dependent manner. The ABTS^+^ scavenging capacity assay is another indicator of the antioxidant activity. AQFE at 500 μg/mL showed similar ABTS^+^ scavenging capacity as vitamin C (50 μg/mL) and trolox (50 μg/mL) (Figure 4B). The IC_50_ of AQFE was 160.79 ± 4.63 μg/mL. The results clearly demonstrated antioxidant capability of AQFE. To measure the reducing activity of AQFE, different concentrations of AQFE (25–500 μg/mL) or trolox (50 μg/mL) were used. AQFE at 500 μg/mL exhibited similar reducing capacity as trolox (Figure 4C), indicating its reducing power. The IC_50_ value of AQFE was compared to some pure antioxidant chemicals used commercially, including ascorbic acid and trolox. As shown in Figure 4, although the antioxidant capacity of AQFE was weaker than those of the pure antioxidants, it was still relatively strong compared to many edible vegetables and components of Chinese medicine [33].

The intracellular antioxidative actions of AQFE were evaluated. After exposure to advanced glycation end products for 72 h, a remarkable increase in intracellular ROS production was observed (Figure 4D). Such AGE-BSA induced intracellular ROS was significantly reduced by pre-treatment with AQFE in a dose dependent manner. At concentrations of 20, 50 and 100 μg/mL, AQFE suppressed ROS by 18.6%, 36.6% and 88.3%, respectively. These data demonstrate that AQFE has a protective effect on HDF cells by attenuating AGE-induced oxidative stress.

### 3.4. The Effect of AQFE on Cellular Senescence

**Figure 5:**
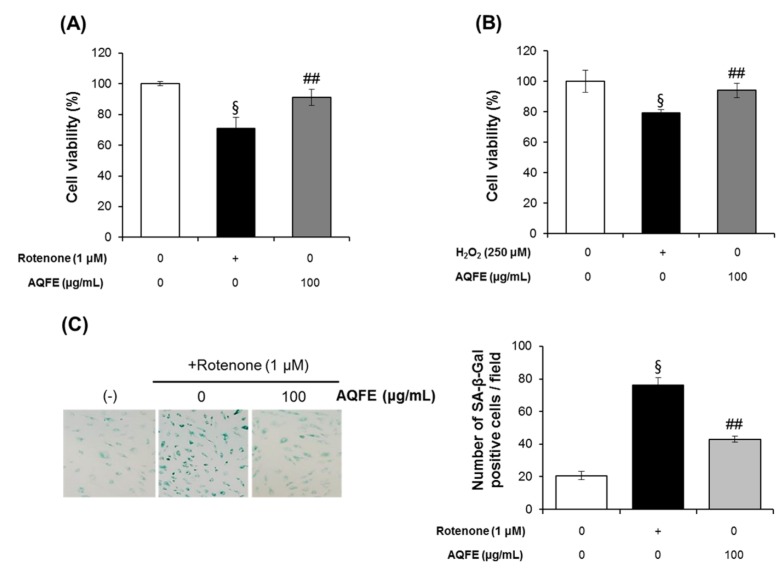

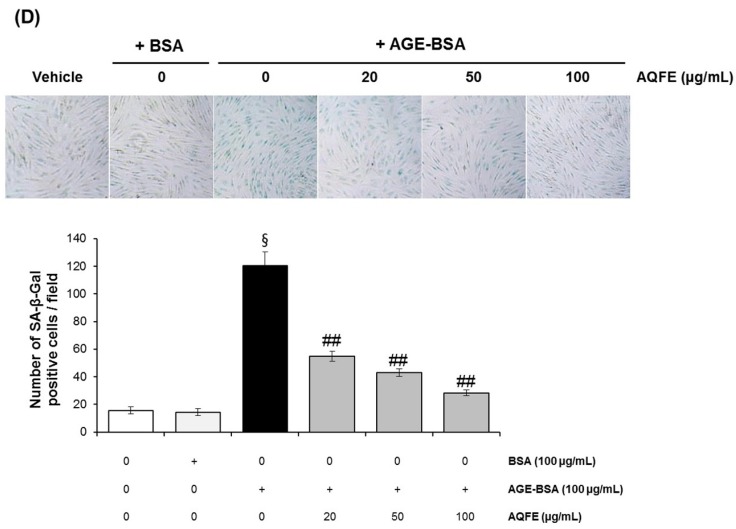
Effect of AQFE treatment on viability against premature senescence induced by oxidative stress to HDFs. AQFE inhibited (**A**) rotenone or (**B**) H_2_O_2_-induced cytotoxicity measured with MTT assays. (**C**) SA-β-gal staining of human dermal fibroblast cells after exposure to rotenone on untreated control, cells treated with rotenone and cells treated with rotenone + AQFE 100 µg/mL. The number of SA-β-gal positive cells per field was calculated using Image J software. Data are mean ± standard deviation. ^§^ *p* < 0.01 compared with the vehicle-treated group; ^##^ *p* < 0.01 compared with the rotenone or H_2_O_2_-treated group (*n* = 3). (**D**) SA-β-gal staining of human dermal fibroblast cells after exposure to advanced glycation end products on untreated batch, treated with BSA, treated with AGE-BSA, treated with AGE-BSA + AQFE 20 µg/mL, treated with AGE-BSA + AQFE 50 µg/mL and treated with AGE-BSA + AQFE 100 µg/mL. Data are mean ± standard deviation. ^§^ *p* < 0.01 compared with the vehicle-treated group; ^#^ *p* < 0.05 compared with the MG-treated group (*n* = 3). The results were confirmed by eight independent experiments.

Oxidative stress is the main cause of cellular senescence. Rotenone was chosen as a senescence-inducing agent because it acts on mitochondrial complex I, leading to increased levels of intracellular ROS and mimicking the physiological process of ROS induced damage that is hypothesized to underlie the aging process [34,35]. Cellular senescence induced by H_2_O_2_ has also been widely used to evaluate the antiaging effects of test materials [36]. H_2_O_2_ can induce oxidative stress in cells, which may involve the overproduction and accumulation of oxygen free radicals [37]. Figure 5A,B show the effect of AQFE on cell viability under premature senescence in HDFs following rotenone or H_2_O_2_ treatment. HDFs were pretreated with 100 μg/mL AQFE for 2 h and then stimulated with 1 μM rotenone or 250 μM H_2_O_2_. Cell viability was analyzed after 72 h. As expected, AQFE pretreatment attenuated both the rotenone and H_2_O_2_-induced decrease in cell viability. Rotenone and H_2_O_2_-treated cells showed a 29.17% ± 7.47% and 20.71% ± 2.11% reduction in cell viability compared with untreated cells, respectively. However, AQFE pretreated cells showed only 8.95% ± 5.28% and 5.97% ± 4.78% reductions compared with untreated cells, respectively. The present study also assayed for the presence of SA-β-galactosidase activity to investigate whether AQFE affects oxidative stress-induced senescence, which would contribute to its protective effect against ROS-mediated cell damage. Rotenone treatment increased the number of SA-β-galactosidase-positive cells to 76.33% ± 4.51% compared with the control. However, only 43.00% ± 1.73% of the cells pretreated with AQFE were SA-β-galactosidase-positive following treatment with Rotenone (Figure 5C). These data indicated that AQFE has the potential to inhibit cellular senescence by oxidative stress in HDF cells.

We observed that AGEs significantly induced intracellular ROS in NHFs (Figure 4D). Therefore, we also investigated whether AGE-BSA induced a senescent phenotype in HDFs. In AGE-BSA-treated HDFs, the ratio of SA-β-gal-positive HDFs was significantly increased compared to control HDFs. The addition of AQFE (20–100 µg/mL) significantly decreased the number of cells positive for SA-β-gal in a dose-dependent manner (Figure 5D). The results suggested that AGEs-induced HDF aging could be significantly attenuated by AQFE.

### 3.5. Anti-Skin Aging Properties of AQFE in Vivo

**Figure 6:**
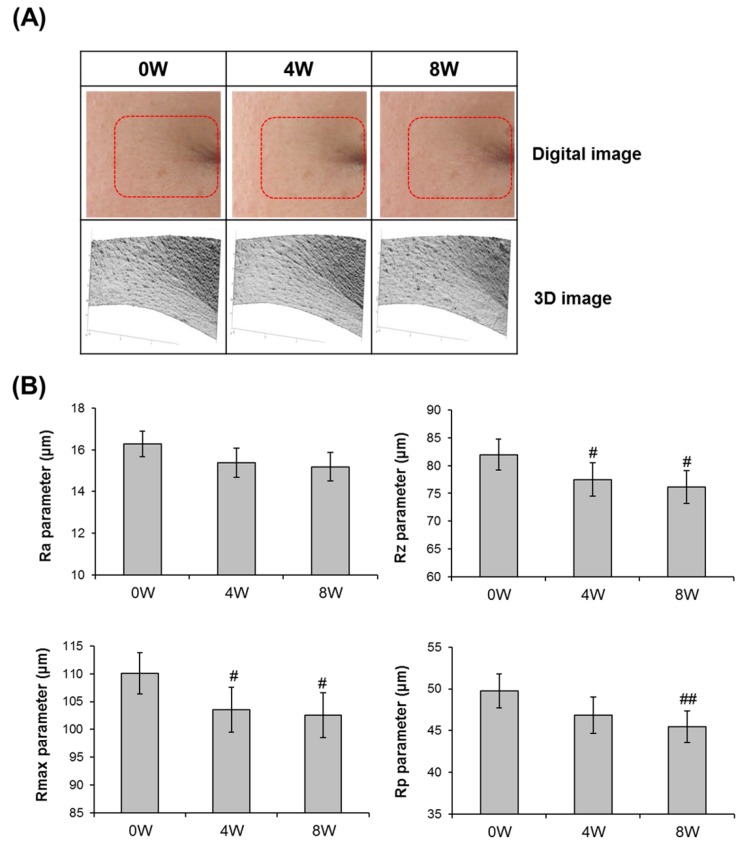
Visual improvement of wrinkles in the crow’s feet area with AQFE (0.5%) application for eight weeks. (**A**) Skin wrinkle images in crow’s feet following application of the test product for eight consecutive weeks (Subject No. 15) (upper; Digital image, lower; 3D image). (**B**) Changes of skin wrinkle parameters in crow’s feet following application of test product for eight consecutive weeks. (Mean ± SEM. ^#^ *p* <0.05; ^##^ *p* < 0.01 *versus* before treatment (0 W)). Ra: arithmetic average roughness; Rz: average roughness; Rmax: maximum roughness; Rp: smoothness depth.

A recent study reported that inhibition of AGE formation is involved in the anti-skin aging effect [38]. Therefore, based on the *in vitro* activities in live human explants, the efficacy of the extract as an anti-skin aging agent was also clinically examined. A formulation containing 0.5% AQFE was applied to the periorbital region of 17 volunteers over eight weeks. Our analysis of the crow’s feet wrinkle showed a decrease in the depth of deep furrows in RI treated with AQFE cream over an eight-week period (*p* < 0.05) (Figure 6).

Skin wrinkles on the test site (crow’s feet area in subjects) were evaluated using 3D Image Analysis GFM PRIMOS (GFMesstechnik GmbH, Germany). The representative data for each group are presented as Ra, Rz, Rmax, and Rp. As shown in Figure 6, compared to before treatment, the means of Rz, Rmax, and Rp of in the AQFE-treated group were significantly improved at eight weeks. The value of Ra, Rz, Rmax, and Rp were decreased by 6.72%, 6.85%, 7.02% and 8.70%, respectively, at eight weeks compared to before treatment. These findings indicate that AQFE exerts an antiwrinkle effect.

## 4. Discussion

AGEs are considered to be markers of various diseases, such as cataract, arteriosclerosis, renal failure, and Alzheimer’s disease [39]. Other than glycation, alterations in extracellular matrix content play key roles in aging. As the process of aging progresses, extracellular matrix including collagen, elastin and fibers undergo lysis and become thinner. We applied AQFE to an AGEs induced-skin aging model to confirm its potential use as an anti-skin aging ingredient.

The model of living human skin explants associated with histology enable the monitoring of early pharmacological activities and the visualization in a few days of reactions that normally unfold over the long term. Glycation was induced by methylglyoxal over a short period of time with the production of AGEs (CML). Fibrillin-1, a glycoprotein that is a component of a very sensitive part of the elastic network (oxytalan fibers), appears to be a very good marker for glycation activity because it is rapidly altered by the MG in this model. AQFE, tested for its potential antiglycation properties, demonstrated the ability to prevent glycation *ex vivo*. When introduced in culture medium and applied topically, AQFE completely inhibited the glycant properties of methylglyoxal. CML was not observed and the fibrillin-1 network was not altered. The beneficial effects of AQFE may be applied to the prevention or management of AGE-mediated pathologies, targeting the biochemical and cellular bases of skin aging in a pleiotropic and complementary manner. For these reasons, further studies should focus on the outcome of investigating the effects in humans.

Glycated proteins are commonly formed by a non-enzymatic reaction between the interaction of reducing sugars (fructose and glucose) and amino groups of proteins through a nucleophilic addition with the formation of Schiff bases [40]. The unstable Schiff bases further rearrange to produce the formation of reversible Amadori products (such as fructosamine). Subsequently, the Amadori products further form cross-linked structures termed AGEs which can be classified into two major groups: fluorescent and cross-linking structures (pentosidine, crosslines, and imidazolones) and non-fluorescent and non-crosslinking structure (N^ε^-CML). During the early stages of glycation, Schiff bases are prone to oxidation, generating free radicals and reactive carbonyl groups and forming AGEs. Consequently, reactive oxygen species-mediated reactions cause structural fragmentation and create short-chain carbohydrate intermediates, which then interact sequentially with lysine and arginine residues to produce AGEs [41,42]. Currently, potential antiglycation mechanisms have been proposed such as breaking the cross-linking structures in the formed AGEs, blocking the carbonyl or dicarbonyl groups in reducing sugars, Schiff bases of Amadori products, and inhibiting the formation of late-stage Amadori products [43]. In the present study, we investigated the influence of AQFE extract on the formation of AGEs. The results showed that AQFE efficiently inhibited fluorescent and non-fluorescent AGE formation.

Autooxidation of sugars and of the products from non-enzymatic glycation of proteins is a free radical-mediated reaction that occurs under aerobic conditions. It has been suggested that oxidative stress induced by hyperglycemia is a key factor in the pathogenesis of age-related diseases. It has been reported that many antioxidant-containing foods can scavenge free-radicals generated during the glycation process in addition to preventing self-oxidation in reducing sugars and Amadori products, leading to the inhibition of AGE formation [44]. In fact, free radicals and oxidative steps have long been regarded as crucial factors in the glycation process [45]. In the present study, various methods of accessing antioxidant capacities have been used for AQFE. DPPH is widely used to evaluate the free radical scavenging abilities of phytochemical compounds *in vitro*. The ABTS^+^ assay has been used to assess the capacity of edible plants to scavenge ABTS radicals [46]. FRAP assay is used extensively to evaluate the ability of edible plants to reduce ferric ions, reflecting their ability to decrease reactive oxygen species (ROS) [47]. From the results obtained in the present study, AQFE showed potent antioxidant properties. According to the abovementioned antiglycation mechanisms, AQFE may inhibit AGE formation by scavenging free radicals formed *in vitro* by the auto-oxidation of sugars and/or oxidative degradation of Amadori products.

Oxidative damage caused by ROS is one of the key sources of cellular senescence [48]. ROS are formed as a natural by-product of normal oxygen metabolism and have important roles in cell signaling and homeostasis [49]. Senescence occurs when there is an imbalance between ROS levels and the antioxidant defense system. When ROS production increases and/or ROS elimination is downregulated, there is an increase in ROS that results in oxidative damage to cellular function and structure [50]. In the present study, we demonstrated that AQFE had clear protecting effects on HDFs aging induced by AGEs and could obviously decrease the level of intracellular ROS in AGEs-treated HDFs. These results suggested that AQFE was an effective antioxidant on HDFs and had obvious protective effects on HDFs aging induced by ROS.

Glycation occurs when glucose or other endogenous reducing sugars bind nonenzymatically to proteins or lipids. This process may also be amplified by UV exposure, impairing the functioning of biomolecules and eventually leading to less firm and less elastic skin that is more prone to wrinkling. In fact, AGEs accumulate in skin partially by binding with collagen proteins. Collagen fibers maintain skin elasticity in combination with elastin fibers. A collagen fiber may be glycated to AGEs and form cross-links with other fibers, thus reducing elasticity. Glycation of collagen may also cause loss of skin elasticity and wrinkle formation. To check whether AQFE has an anti-skin aging effect on human skin, we performed preliminary clinical tests for skin wrinkling. Our analysis of crow’s feet wrinkles showed that the mean values of skin wrinkle parameters in the AQFE-treated group were significantly improved after 8 weeks. Clinical studies further suggested that treatment with formulations containing AQFE confers anti-skin aging benefits. Additional investigations are needed to further confirm the observed results as well as to elucidate the molecular mechanisms at the base of the biological activity.

AGEs and oxidative stress have been implicated in the pathogenesis of skin disorders or skin ageing. Both are known to interact with each other. Therefore, natural compounds or extracts that possess both antioxidant and antiglycation activities might have great therapeutic potential for treating skin aging and skin disorders. In this aspect, our results suggest that AQFE is a good candidate material to suppress skin ageing that is induced by internal or external stresses. In addition, we found that myo-inositol were included in the ethanol extract of *Akebia quinta* fruit (data not shown). Myo-inositol is present in various fruits such as citrus fruits and beans. It has been reported to have anti-oxidant activity through quenching action on ROS and anti-glycation activity through scavenging of glucose [51]. *Akebia quinta* has been reported to contain chlorogenic acid and triterpenoid saponins such as oleanolic acid and hederagenin. Chlorogenic acid and triterpenoid saponins were reported to contribute antioxidant and antiglycation activity [24,52,53,54]. Therefore, although it needs further study, there is the possibility that myo-inositol and triterpenoid saponins are effective AQFE compounds for suppressing skin aging.

## 5. Conclusions

Our findings demonstrate that AQFE protects against glucose-mediated glycation *in vitro*. Additionally, AQFE clearly prevented glycation in human skin explants. AQFE has the potential to inhibit cellular senescence by oxidative stress in HDF cells and no skin primary irritation in humans. In addition, the efficacy of AQFE as an anti-skin aging agent was clinically validated. We suggest that AQFE can be used as a possible anti-skin aging agent, as it prevents oxidative stress and other complications associated with AGEs formation.
